# Supplementary material for: Polyketide synthase gene clusters in the ericoid mycorrhizal fungus Oidiodendron maius: genome-wide prediction, expression profiling, and functional characterization of knockout mutants
Source: Front Plant Sci. 2026 Sep 16;17:1924430. doi: 10.3389/fpls.2026.1924430 (PMC13623903; doi:10.3389/fpls.2026.1924430)
Supplement: Supplementary file 1 [file SupplementaryFile1.zip › Supplementary Materials/Supplementary Folder 1.zip!/Oidma1_AssemblyScaffolds_Repeatmasked/knownclusterblast/cluster4/jgi.p_Oidma1_21939_mibig_hits.html]

| MIBiG Protein | Description | MIBiG Cluster | MiBiG Product | % ID | % Coverage | BLAST Score | E-value |
| --- | --- | --- | --- | --- | --- | --- | --- |
| EAU38788.1 | conserved\_hypothetical\_protein | BGC0000161 | Terrein\_biosynthetic\_gene\_cluster | 67 | 101.180438449 | 847 | 6.8e-246 |
| CBW45655.1 | putative\_oxygenase | BGC0000952 | Pristinamycin\_biosynthetic\_gene\_cluster | 31 | 60.2023608769 | 165 | 2.7e-40 |
| ADE34483.1 | SsfO2 | BGC0000269 | SF2575\_biosynthetic\_gene\_cluster | 27 | 79.258010118 | 163 | 5.9e-40 |
| AEI98648.1 | CtcE | BGC0000209 | Chlortetracycline\_biosynthetic\_gene\_cluster | 29 | 78.7521079258 | 159 | 1.1e-38 |
| CAK50777.1 | oxygenase | BGC0000247 | Mithramycin\_biosynthetic\_gene\_cluster | 29 | 60.5396290051 | 157 | 4.2e-38 |
| CAA07757.1 | oxygenase | BGC0000246 | Mithramycin\_biosynthetic\_gene\_cluster | 29 | 60.5396290051 | 157 | 4.2e-38 |
| CAE17550.1 | oxygenase | BGC0000210 | Chromomycin\_A3\_biosynthetic\_gene\_cluster | 28 | 62.7318718381 | 156 | 1.2e-37 |
| AFU65903.1 | DacO2 | BGC0000216 | Dactylocycline\_biosynthetic\_gene\_cluster | 29 | 60.2023608769 | 151 | 3e-36 |
| AAZ78335.1 | OxyL | BGC0000254 | Oxytetracycline\_biosynthetic\_gene\_cluster | 28 | 80.9443507589 | 149 | 8.8e-36 |
| AEF32090.1 | monooxygenase | BGC0001333 | BE-54017\_biosynthetic\_gene\_cluster | 29 | 63.2377740304 | 144 | 3.7e-34 |
| AKD43496.1 | Monooxygenase | BGC0001409 | Dutomycin\_biosynthetic\_gene\_cluster | 28 | 60.0337268128 | 142 | 1.4e-33 |
| CAM34363.1 | putative\_FAD-binding\_monooxygenase,\_PheA/TfdB\_family | BGC0000242 | Lysolipin\_biosynthetic\_gene\_cluster | 30 | 62.7318718381 | 138 | 2.7e-32 |
| AEO12714.1 | flavin\_monooxygenase | BGC0001334 | Cladoniamide\_biosynthetic\_gene\_cluster | 29 | 61.3827993255 | 137 | 5.9e-32 |
| AKQ20687.1 | monooxygenase | BGC0001337 | Lazarimide\_biosynthetic\_gene\_cluster | 28 | 60.8768971332 | 132 | 1.5e-30 |
| AGD80608.1 | monooxygenase | BGC0000394 | Naphthyridinomycin\_biosynthetic\_gene\_cluster | 29 | 59.8650927487 | 126 | 1.4e-28 |
| CAO98853.1 | monooxygenase\_AufJ | BGC0000023 | Aurafuron\_biosynthetic\_gene\_cluster | 28 | 60.2023608769 | 124 | 5.2e-28 |
| AAD13534.1 | oxygenase\_homolog | BGC0000239 | Landomycin\_biosynthetic\_gene\_cluster | 26 | 68.1281618887 | 120 | 7.5e-27 |
| ACN64853.1 | PokO2 | BGC0001061 | Polyketomycin\_biosynthetic\_gene\_cluster | 27 | 60.8768971332 | 120 | 7.5e-27 |
| CAD19095.1 | cytochrome\_P450\_dependent\_monooxygenase | BGC0000153 | Stigmatellin\_biosynthetic\_gene\_cluster | 27 | 59.1905564924 | 120 | 7.5e-27 |
| CAK50794.2 | oxygenase | BGC0000247 | Mithramycin\_biosynthetic\_gene\_cluster | 29 | 60.370994941 | 119 | 1.3e-26 |
| ABO15838.1 | FAD-dependent\_monooxygenase | BGC0000130 | Pyrrolomycin\_biosynthetic\_gene\_cluster | 29 | 59.6964586847 | 119 | 1.3e-26 |
| EHM27503.1 | monooxygenase\_FAD-binding | BGC0000235 | Kiamycin\_biosynthetic\_gene\_cluster | 25 | 72.849915683 | 119 | 1.3e-26 |
| ADB02842.1 | AzicO4 | BGC0000202 | Azicemicin\_biosynthetic\_gene\_cluster | 25 | 74.3676222597 | 117 | 4.9e-26 |
| AEE65484.1 | monooxygenase | BGC0000223 | Fluostatin\_biosynthetic\_gene\_cluster | 27 | 61.2141652614 | 117 | 6.4e-26 |
| CBF80483.1 | FAD-dependent\_monooxygenase\_(Eurofung) | BGC0000959 | Aspyridone\_biosynthetic\_gene\_cluster | 27 | 72.6812816189 | 114 | 3.2e-25 |
| WP\_030269258.1 | monooxygenase | BGC0001308 | Simocyclinone\_D9\_/\_simocyclinone\_D10\_/\_simocyclinone\_D11 | 26 | 59.3591905565 | 113 | 9.2e-25 |
| WP\_030269274.1 | oxidoreductase | BGC0001308 | Simocyclinone\_D9\_/\_simocyclinone\_D10\_/\_simocyclinone\_D11 | 27 | 73.3558178752 | 113 | 1.2e-24 |
| AKD43495.1 | Monooxygenase | BGC0001409 | Dutomycin\_biosynthetic\_gene\_cluster | 27 | 64.0809443508 | 112 | 1.6e-24 |
| ACP19351.1 | SaqE | BGC0000267 | Saquayamycin\_Z\_/\_galtamycin\_B\_biosynthetic\_gene\_cluster | 26 | 59.5278246206 | 112 | 2e-24 |
| CBH32810.1 | putative\_oxygenase | BGC0000263 | Ravidomycin\_biosynthetic\_gene\_cluster | 28 | 61.0455311973 | 112 | 2e-24 |
| AGZ15446.1 | putative\_polyketide\_oxygenase/hydroxylase | BGC0001036 | Polyoxypeptin\_biosynthetic\_gene\_cluster | 27 | 61.8887015177 | 111 | 2.7e-24 |
| AAK57522.1 | PgaE | BGC0000262 | Rabelomycin\_biosynthetic\_gene\_cluster | 26 | 59.6964586847 | 111 | 3.5e-24 |
| ACN64854.1 | PokO4 | BGC0001061 | Polyketomycin\_biosynthetic\_gene\_cluster | 27 | 63.2377740304 | 110 | 7.8e-24 |
| ABC02791.1 | putative\_FAD-monooxygenase | BGC0000809 | AT2433\_biosynthetic\_gene\_cluster | 27 | 61.0455311973 | 110 | 7.8e-24 |
| AAM97364.1 | RubN | BGC0000266 | Rubromycin\_biosynthetic\_gene\_cluster | 25 | 72.1753794266 | 110 | 7.8e-24 |
| AAL15585.1 | Sim7 | BGC0000270 | Simocyclinone\_biosynthetic\_gene\_cluster | 28 | 60.5396290051 | 110 | 7.8e-24 |
| ADE22302.1 | putative\_FAD-binding\_monooxygenase | BGC0000279 | Xantholipin\_biosynthetic\_gene\_cluster | 24 | 64.0809443508 | 108 | 3.9e-23 |
| AAK06782.1 | putative\_oxygenase\_SimA7 | BGC0001072 | Simocyclinone\_biosynthetic\_gene\_cluster | 26 | 59.5278246206 | 107 | 6.6e-23 |
| AEF32100.1 | RebC-like\_monooxygenase | BGC0001333 | BE-54017\_biosynthetic\_gene\_cluster | 28 | 60.5396290051 | 106 | 1.1e-22 |
| CBH32087.1 | putative\_FAD-dependent\_monooxygenase | BGC0000211 | Chrysomycin\_biosynthetic\_gene\_cluster | 26 | 61.3827993255 | 101 | 2.8e-21 |
| AAM33673.1 | putative\_FAD-dependent\_monooxygenase\_GrhO6 | BGC0000230 | Griseorhodin\_biosynthetic\_gene\_cluster | 26 | 59.3591905565 | 101 | 4.7e-21 |
| ABP54640.1 | monooxygenase,\_FAD-binding | BGC0000241 | Lomaiviticin\_biosynthetic\_gene\_cluster | 27 | 60.2023608769 | 99 | 1.8e-20 |
| AKQ20690.1 | RebC-like\_monooxygenase | BGC0001337 | Lazarimide\_biosynthetic\_gene\_cluster | 26 | 64.2495784148 | 97 | 6.8e-20 |
| AHZ61860.1 | Lom26 | BGC0000240 | Lomaiviticin\_biosynthetic\_gene\_cluster | 26 | 59.5278246206 | 96 | 1.5e-19 |
| ADB02837.1 | AzicO3 | BGC0000202 | Azicemicin\_biosynthetic\_gene\_cluster | 33 | 32.5463743676 | 95 | 2e-19 |
| AAK06789.1 | putative\_oxygenase\_SimA8 | BGC0001072 | Simocyclinone\_biosynthetic\_gene\_cluster | 31 | 33.8954468803 | 95 | 2e-19 |
| AKG47162.1 | HysC | BGC0001223 | Hydroxysporine\_biosynthetic\_gene\_cluster | 24 | 61.8887015177 | 95 | 2.6e-19 |
| ACP19358.1 | SaqM | BGC0000267 | Saquayamycin\_Z\_/\_galtamycin\_B\_biosynthetic\_gene\_cluster | 25 | 60.0337268128 | 94 | 4.4e-19 |
| AAP69582.1 | putative\_FAD-dependent\_oxygenase | BGC0000226 | Gilvocarcin\_biosynthetic\_gene\_cluster | 31 | 33.0522765599 | 94 | 4.4e-19 |
| ABP54639.1 | monooxygenase,\_FAD-binding | BGC0000241 | Lomaiviticin\_biosynthetic\_gene\_cluster | 25 | 59.3591905565 | 92 | 1.7e-18 |
| AHZ61851.1 | Lom17 | BGC0000240 | Lomaiviticin\_biosynthetic\_gene\_cluster | 28 | 51.2647554806 | 90 | 6.4e-18 |
| AHZ61850.1 | Lom16 | BGC0000240 | Lomaiviticin\_biosynthetic\_gene\_cluster | 29 | 32.0404721754 | 86 | 9.2e-17 |
